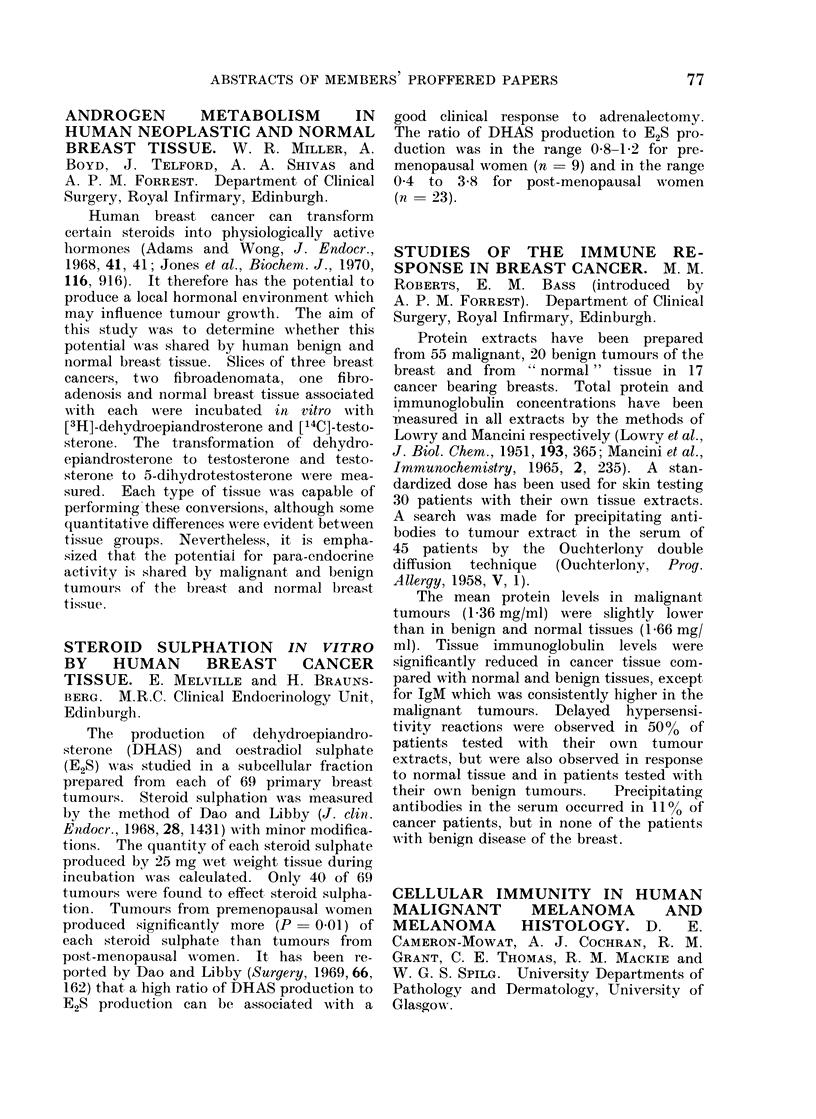# Androgen metabolism in human neoplastic and normal breast tissue.

**DOI:** 10.1038/bjc.1973.76

**Published:** 1973-07

**Authors:** W. R. Miller, A. Boyd, J. Telford, A. A. Shivas, A. P. Forrest


**ANDROGEN METABOLISM IN HUMAN NEOPLASTIC AND NORMAL BREAST TISSUE.** W. R. MILLER, A. BOYD, J. TELFORD, A. A. SHIVAS and A. P. M. FORREST. Department of Clinical Surgery, Royal Infirmary, Edinburgh.

Human breast cancer can transform certain steroids into physiologically active hormones (Adams and Wong, *J. Endocr.*, 1968, **41**, 41; Jones *et al.*, *Biochem. J.*, 1970, **116**, 916). It therefore has the potential to produce a local hormonal environment which may influence tumour growth. The aim of this study was to determine whether this potential was shared by human benign and normal breast tissue. Slices of three breast cancers, two fibroadenomata, one fibroadenosis and normal breast tissue associated with each were incubated *in vitro* with [$^3$H]-dehydroepiandrosterone and [$^{14}$C]-testosterone. The transformation of dehydroepiandrosterone to testosterone and testosterone to 5-dihydrotestosterone were measured. Each type of tissue was capable of performing these conversions, although some quantitative differences were evident between tissue groups. Nevertheless, it is emphasized that the potential for para-endocrine activity is shared by malignant and benign tumours of the breast and normal breast tissue.